# Punicalagin Mollifies Lead Acetate-Induced Oxidative Imbalance in Male Reproductive System

**DOI:** 10.3390/ijms17081269

**Published:** 2016-08-11

**Authors:** Faiza Rao, Yiwen Zhai, Fei Sun

**Affiliations:** 1 Institute of Immunology and CAS (Chinese Academy of Sciences) Key Laboratory of Innate Immunity and Chronic Disease, Innovation Center for Cell Biology, School of Life Sciences and Medical Center, University of Science and Technology of China, Hefei 230027, Anhui, China; rao.faiza@yahoo.com (F.R.); zhyiwen@mail.ustc.edu.cn (Y.Z.); 2 Hefei National Laboratory for Physical Sciences at Microscale, Hefei 230027, Anhui, China; 3 International Peace Maternity & Child Health Hospital. School of Medicine, Shanghai Jiaotong University, Shanghai 200025, China

**Keywords:** fertility, oxidative stress, punicalagin, lead acetate, testicular damage

## Abstract

Punicalagin (PU) is a known antioxidant. The present study examined PU to protect against lead-induced oxidative stress (OS) testicular damage in mice. Significant increase in lipid peroxidation (LPO) after intraperitoneal injection of lead acetate (LA) indicated enormous generation of reactive oxygen species (ROS). Lead-induced OS has a direct effect on the differentiation of spermatogonial cells, showing a significant decline in sperm count. Supplementation of PU significantly changes values of LPO and glutathione (GSH) with a concomitant increase in sperm count, a marked decrease in the abnormal sperms, and a decline in the morphologically abnormal sperm population. Moreover, the histopathological evaluation of testes and epididymides showed severe changes in mice treated with LA. PU significantly induced nuclear factor erythroid-2 related factor 2-like 2 (Nrf2) expression and phase II enzymes, and data suggest that PU may inhibit OS through Nrf2 activation. The fertility test proved that PU might play an important role in male infertility treatment, especially in the type of infertility induced by OS.

## 1. Introduction

Lead compound toxicity has been studied in different human diseases. It is used for various industry and household products [1,2]. It has been previously studied that, compared to other body organs, testes are more sensitive to oxidative stress (OS) and are less defendable against OS due to the presence of high polyunsaturated fatty acids. Accumulated evidence has revealed that testicular physiology, which is basically characterized by the spermatogenesis process, gets disrupted by reactive oxygen-dependent mechanisms [3]. Lead acetate (LA) elicits toxic pathological changes in the testis, leading to atrophy of the organ [4,5,6,7]. Seminal cytology of lead-intoxicated animals normally depicts asthenospermia, hypospermia, teratospermia and remarkable changes in sperm count [8]. Studies in human and animals previously proved that sperm motility, DNA damage and sperm shape are affected by lead [9,10]. Previously, it was known that OS is induced by LA. Every organ has a different ability to bear oxidative stress. The testes are receptive to OS, so lead-induced OS is obvious in the testes. However, it has been shown that because the testis in particular readily succumbs to OS [11], reactive oxygen species (ROS) gather in the reproductive system, and with the triggering of lipid peroxidation (LPO), OS is induced [12,13]. It is well documented that ROS generation results in the activation of LPO. Many scientists have used different antioxidants, including vitamin C [14], vitamin E [15], curcumin [16], pomegranate juice (PJ) [17], and many others during their investigations. The beneficial effects of antioxidants on heavy metal-induced toxicity or OS have been documented previously [18]. Previous studies revealed that PJ contains certain constituents that appear to have beneficial therapeutic properties, including antioxidative effects [19,20]. The extracts contain a number of polyphenols, including anthocyanins, minor flavonoids and punicalagin (PU), which is the most important member of the ellagitannins family. PU is the largest polyphenol among the pomegranate ellagitannins and it is responsible for most of the antioxidant activity of the PJ [21]. PU as a strong antioxidant can also play a vital role in clinical and experimental studies, as other polyphenols have demonstrated their role [22]. In our previous study, we used PU of 98% purity against lipopolysaccharide (LPS)-induced OS testicular damage [23]. However, here LA is used to cause high damage in the testis and we want to evaluate the PU defensive capacity against high oxidative stress-induced damage. A previous study mentioned that pomegranate extracts improve semen quality in infertile men [24]. The biochemical and histopathological effects of daily PU consumption on the OS parameters and sperm concentrations of mice were subsequently evaluated.

## 2. Results

### 2.1. Evaluation of Testicular Oxidative Damage and Antioxidant Enzymes

A significant decrease in the mean testicular weight was observed in the LA-treated group in comparison with the control group and PU + LA groups (Table 1). LA showed a significant increase in the LPO concentration as compared to the control while PU and LA co-administration showed a marked decrease in LPO (Table 1). PU and LA co-administration resulted in an increase in glutathione (GSH) level as compared to the LA group (Table 1). The 8-hydroxy-2′-deoxyguanosine (8-OHdG) values in the PU-treated group and control group were not different from each other. A marked increase of 8-OHdG in the LA group was observed as compared to the PU and LA co-administered group (Table 1).

### 2.2. Testicular Histology

**Figure 1:**
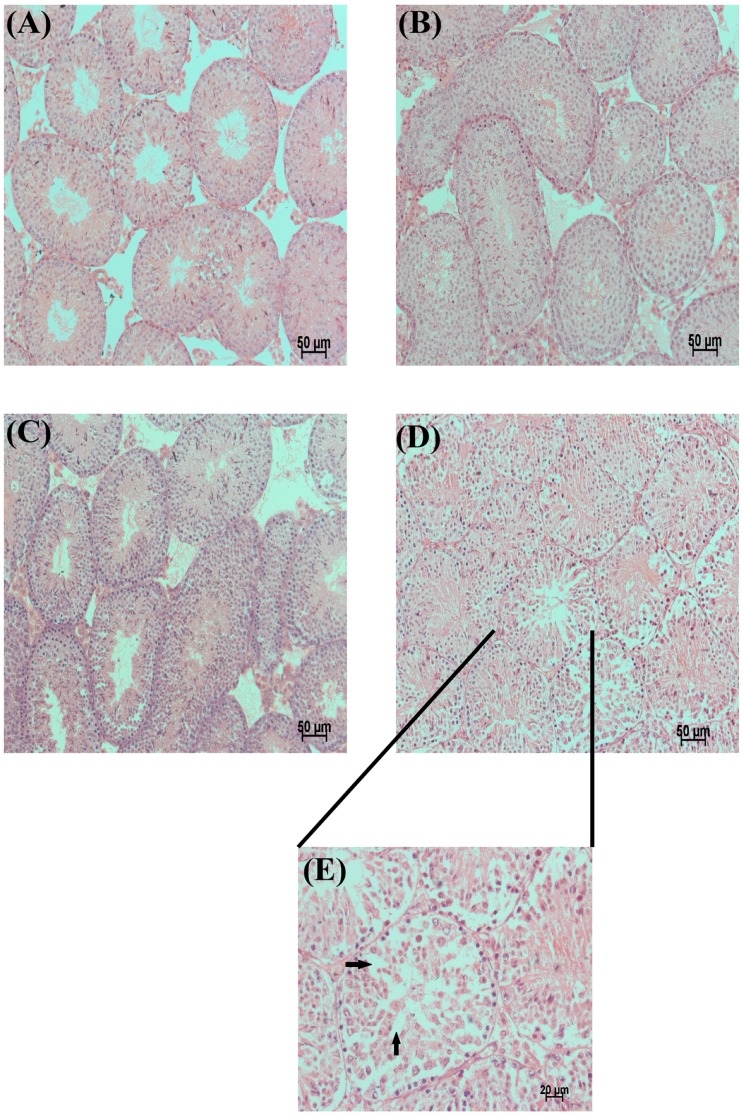
Testes histology. (**A**,**B**) Regular-shaped and normal spermatogenesis observed in control and punicalagin (PU)-injected mice testes; (**C**) Co-injection of PU and lead acetate (LA) protected testes against oxidative injury; normal seminiferous tubules with presence of sperm and less degenerative germ cells could be observed; (**D**) An amount of 100 mg/kg of LA administered intraperitoneally in mice; a single dose causes oxidative stress–induced damage in mice testes. The figure shows degeneration of germ cells, absence of sperm and vacuolation formation in testicular tissue; (**E**) Degeneration of seminiferous epithelium, vacuolization (arrow in figure), disordered epithelium and loss of sperms were evidenced in LA-treated group.

**Figure 2:**
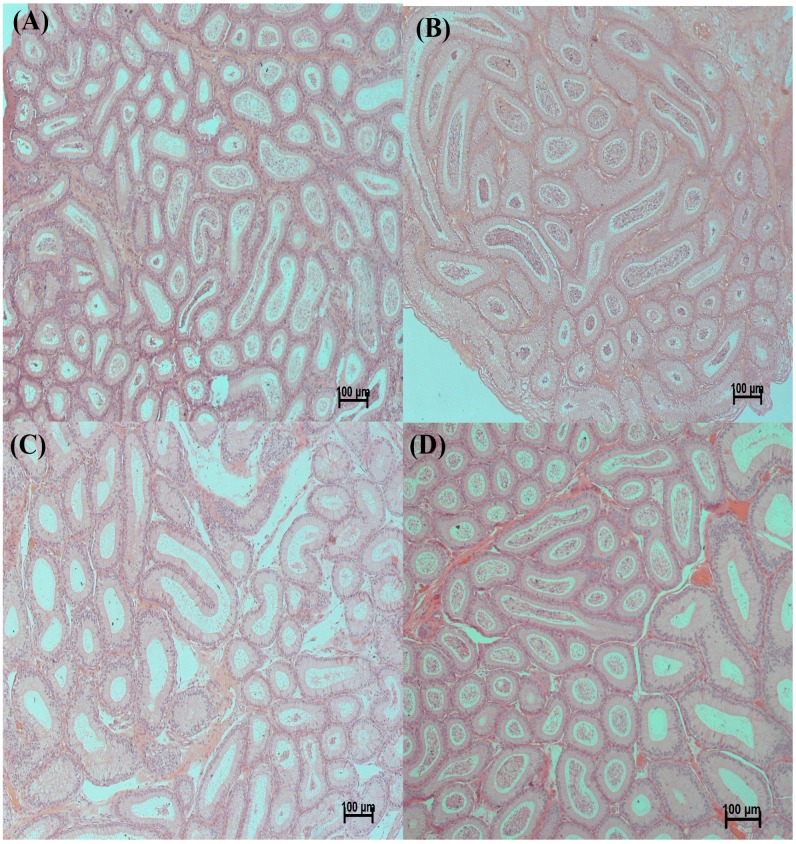
Epididymis histology. (**A**,**B**) Control and PU groups show maximum number of sperms in epididymis; (**C**) LA-induced OS proved damage to sperms as the epididymis shows reduction of sperm count; (**D**) PU + LA co-administration presented increase in number of sperms in epididymis.

In the control and PU groups, the testes contained a well-organized seminiferous epithelium, along with the presence of mature spermatids at the luminal edge, demonstrating the normal process of spermatogenesis (Figure 1A,B). The epididymis of the control and PU groups showed a maximum number of sperms (Figure 2A,B). Some seminiferous tubules showed disorganization of their lining epithelium (Figure 1D,E). Most of the seminiferous tubules of the testes in the LA-treated group showed a complete absence of primary spermatocytes, secondary spermatocytes, spermatids and spermatozoa and a loss of the spermatogenesis process in comparison with the normal structure of the seminiferous tubules in the control mice. The epididymis of the LA group showed a marked reduction of sperms due to its toxicity (Figure 2C). The co-administration of PU and LA resulted in a normal structure of the seminiferous tubules of the testes and normal spermatogenesis (Figure 1C). The epididymis also appeared to have a maximum number of sperms which proved that PU could be used against OS–induced damage to testes and sperms (Figure 2D).

### 2.3. Nuclear Factor Erythroid-2 Factor 2 (Nrf2) Activation and Related Gene Expression

**Figure 3:**
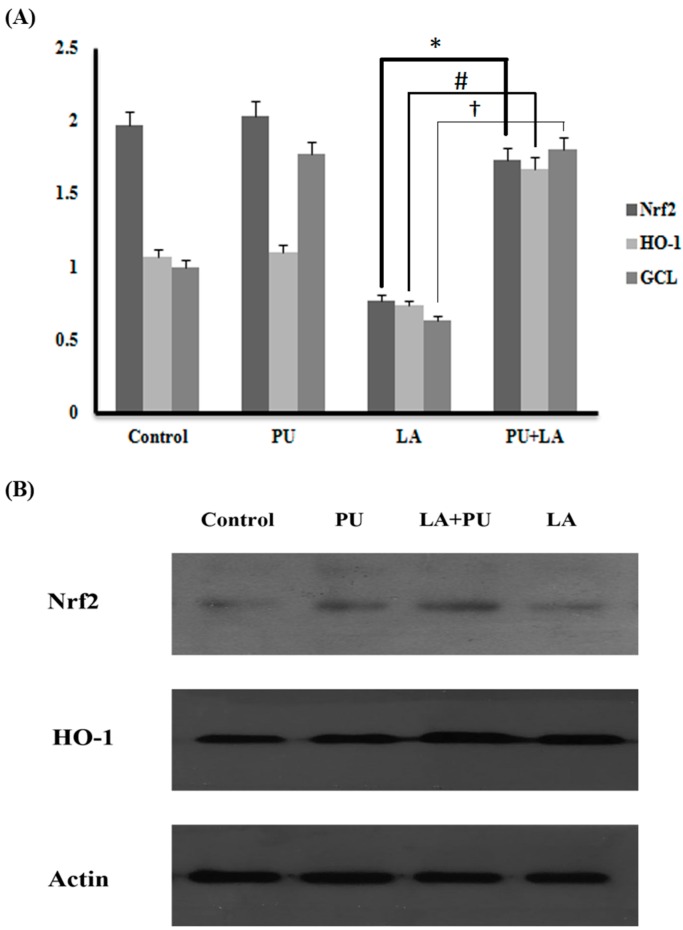
Nuclear factor (erythroid-derived 2)-like 2 (Nrf2) activation. (**A**) Real time reverse transcript-PCR was performed to detect expression of Nrf2 and its target genes heme oxygenase-1 (*HO-1*) and glutamyl-cysteine ligase (*GCL*); (**B**) Western blot analysis results show Nrf2 and HO-1 increase in LA + PU. (*, #, † *p* < 0.0001 values compared for two groups).

The administration of PU in mice was able to induce Nrf2 activation; it was evidenced by the increased expression of Nrf2 target genes heme oxygenase-1 (*HO-1*) and glutamyl-cysteine ligase (*GCL*) (Figure 3A). The Western blot results for Nrf2 and HO-1 in the PU + LA group proved that PU helps in boosting the Nrf2 pathway, which helps to fight against OS damage in the testes (Figure 3B).

### 2.4. Sperm Assessment

**Figure 4:**
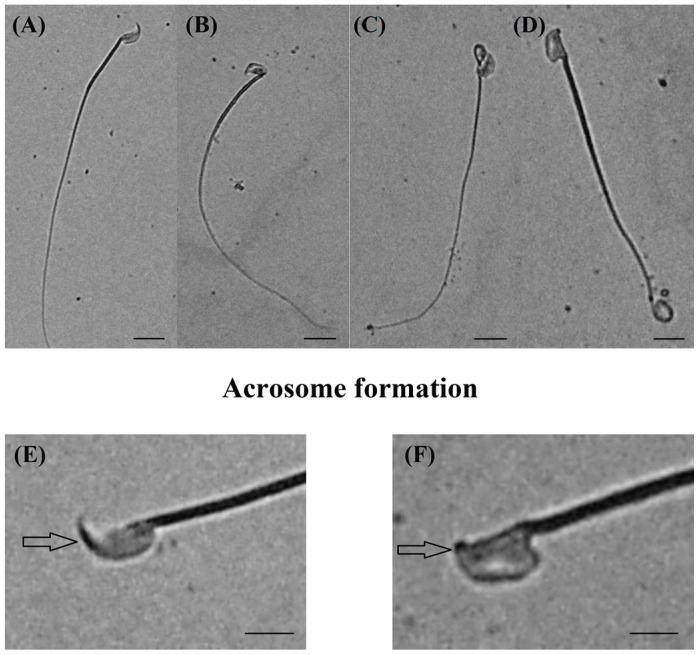
Sperm morphology. (**A**,**E**) Normal sperm, pointed out by arrow showing acrosome formation as dark black color on sperm head; (**B**) abnormal sperm with bent head; (**C**) abnormal sperm with twisted head and neck; (**D**) abnormal sperm morphology with head damage and coiled tail; (**F**) arrow head showing abnormal formation of acrosome.

The sperm count in the LA group was noted to be significantly lower, while those of PU and the PU + LA groups were not significantly different from that of the control. The spermatozoa of the PU + LA group were found to be of significantly better morphology; those of the LA group were of significantly poor morphology (Figure 4) while those of the PU group were not significantly different from those of the control (Table 2).

### 2.5. Fertility Appraisal

The fertility test ended with a remarkable difference in the number of pups of the LA group as compared to the control and PU + LA co-administered groups (Table 3). LA proved its toxicity on the reproductive system. It had an impact on the sperm count and morphology which resulted in a smaller number of pups. The number of LA group pups was also reduced as compared to the control and other groups. Six females were not pregnant at all; these were also not found with the plug, so we concluded that LA-induced OS may also affect sexual behavior due to which, within the exposure time, the males did not engage sexually.

## 3. Discussion

The goal of the present study was to investigate the effect of PU (98% purity) in LA-induced testicular toxicity in mice. Previous studies describe the effect of heavy metals on male fertility [25]. The effect of LA on male reproduction has been documented well in various experimental species. Induced modification in sperm morphology, motility, and count, as well as biochemical disruptions of enzymes with the ROS mechanism are highly affected by this heavy metal exposure [26]. Environmental pollutants such as lead can threaten living creatures in different ways. Due to the increase of lead exposure in the environment, its toxic effect on different organs and their systems has been studied [27]. A direct or indirect effect of a low dose of lead on sexual development and reproduction is documented [28,29]. The study authenticated that the group which received LA (dose of 100 mg/kg) had significantly reduced levels of GSH in the testicular tissue homogenate when compared with control animals, and it had significantly increased LPO contents when compared with control group. These results correlate well with previous investigations [26,30]. A significant increase in levels of LPO activity in the testicular tissue homogenate when compared with the control group verifies the previous studies [31,32,33]. Lead toxicity leads to the degeneration of ROS, including hydroperoxide, singlet oxygen, and hydrogen peroxide, and this leads to the direct depletion of antioxidant reserves as lead has also been shown to suppress blood levels of the antioxidant enzymes superoxide dismutase (SOD) and catalase (CAT) [34,35]. High doses of LA caused a spermicidal effect, resulting in sperm count reduction, which supports the previous studies in which the testicular sperm count is an important indicator of the adverse effect of lead on spermatogenesis, and this can be accounted for by the direct influence of lead on testicular tissues. Sperm abnormality increases with the administration of high doses of lead. It has been reported that OS-induced toxicity disrupts spermatogenesis [36]. Pomegranate’s effect on sperm production in male mice treated with LA has been proved in previous studies. However, we used 98% pure PU and proved that PU alone could have great beneficial effects on sperm production. In the current study, the administration of PU (9 mg/kg) for four weeks with the co-administration of LA showed a significant increase in the levels of GSH and also a significant decrease in the LPO levels when compared with the LA-treated group. The antioxidant enzymes SOD and CAT also decreased in the LA group. Here it can be concluded that lead implicates testicular LPO due to free radical induction. PU decreased LPO and GSH increased due to its powerful antioxidant property. It has previously been documented that nuclear factor Nrf2 is bound to Kelch-like ECH-associated protein-1 (Keap1) in the cytoplasm under normal conditions. Under OS or other potentially damaging stimuli, Nrf2 is released from Keap1 and translocates to the nucleus, where it binds to antioxidant response element (ARE) sequences [37]. Nrf2’s protective potential role against different types of toxicity has also been studied [38]. In this study we tested whether Nrf2 was involved in the damage protection of PU in LA-induced OS testicular damage. We found that Nrf2 increased significantly in the testicular tissue upon PU treatment. Other studies showed some polyphenols also activate Nrf2 [19]. Since Nrf2 is a very important endogenous antioxidant, it is possible that increasing Nrf2 activity might mediate PU-induced attenuation of OS in the testes. To prove our findings, we organized a fertility test. This fertility test proved that maximum damage to sperm morphology resulted in a smaller number of pups with weight difference as compared to controls. PU and LA co-administration significantly increased the sperm count and improved sperm morphology which led to an increase in the number of pups and normal weight. The reason for the smaller number of pups in the LA group could be acrosome damage, which renders the sperm unable to fertilize the egg. The fertility test in this study can conclude that lead exposure decreased libido in male mice. Nonetheless, additional studies are recommended on this subject before clinical application can be endorsed.

## 4. Materials and Methods

### 4.1. Study Design

Thirty-two (weighing between 25–28 g) adult male ICR mice (*Mus musculus*) were used for this study. Mice were obtained from the animal house of University of Science & Technology of China (USTC), Hefei, China. Mice were kept in plastic cages; four mice in each cage. Soft crushed wood shaving was used to cover the floor of all cages. The cages were kept clean, and mice were allowed an ad libitum approach to food and water. Mice were managed under standard laboratory conditions (22–24 °C, 12 h light/12 h dark cycle). All animal-based examinations were designed and performed with the recommendations in the Guide for the Care and Use of Laboratory Animals of National Institutes of Health and received approval from institutional review boards of the University of Science and Technology of China (USTC). Mice were randomly divided into four groups (eight ICR mice in each group) as follows: Control group mice were provided with water and fed with normal diet. PU group animals received PU (9 mg/kg/day) orally by gavage daily for four weeks. LA group mice were injected with LA (100 mg/kg intraperitoneally) for four weeks. PU + LA co-administered animals received PU (9 mg/kg) orally by gavage concurrently injected with LA (100 mg/kg intraperitoneally). All doses were prepared in water. Male treated mice were exposed to normal female mice on the last day of treatment.

### 4.2. Histopathological Examination of Testes

Testes were weighed with a digital balance. Small pieces of testes fixed in Bouin’s solution and 70% ethanol, were taken and dehydrated in graded ethanol, embedded in paraffin, and sectioned (5 μm thickness). Hematoxylin-eosin was used to stain the sections and they were observed under light microscope. Epididymides of mice from all groups were collected and fixed in Bouin’s solution for histological study.

### 4.3. LPO, GSH and Endogenous Antioxidants Evaluation

LPO in the testes homogenate was measured using a LPO Assay Kit (NJJCBio Nanjing, China). The testes were homogenized on ice using 0.2 g tissue in 1.8 mL physiological saline, centrifuged at 2500 rpm for 10 min (ThermoFisher Scientific Heraeus Pico 17, Osterode, Germany). The supernatant was collected. Then 200 µL was used in 96 well plate to detect optical density (OD) at 586 nm (U-3900/3900H HITACHI, Eppendorf AG, Hamburg, Germany). GSH in the testes homogenate was measured using a GSH Assay Kit (NJJCBio). The testes were homogenized on ice in 9 mL of normal saline per gram tissue, centrifuged at 2500 rpm (ThermoFisher Scientific Heraeus Pico 17, Osterode, Germany) for 10 min at 4 °C. The supernatant was used for the assay, according to the instructions in the kit and absorbance of each sample at 420 nm in a glass cuvette was measured.

### 4.4. The 8-OH-dG Assay

DNA oxidative damage enzyme-linked immunosorbent assay (ELISA) kit (Cayman Chemical, Ann Arbor, MI, USA) was used to measure damage in all groups. The experiment was carried out according to the manual provided by the company. Shortly, testes samples were thawed and 5 mL of homogenization buffer per gram of tissue was added. They were centrifuged at 1000× *g* for 10 min and the supernatant was purified using a commercially available DNA extraction kit (Sangon, Shanghai, China). DNA was digested following the manufacturer’s instructions. One unit of alkaline phosphatase was added per 100 µg of DNA and incubated at 37 °C for 30 min. It was then boiled for 10 min. Reagents were added in 9-well plates along with DNA samples and incubated for 18 h at 4 °C. After adding other reagents like EIA buffer, DNA Oxidative Damage EIA standard, samples, DNA Oxidative Damage AChE Tracer and DNA Oxidative Damage EIA Monoclonal Antibody, the plate was read at a wavelength between 405–420 nm.

### 4.5. Western Blot

For Western blot the samples were lysed with Western lysis buffer (Radioimmunoprecipitation assay (RIPA) buffer, inhibitor cocktail and phenylmethylsulfonyl fluoride (PMSF). They were homogenized and centrifuged at 14,000 rpm (ThermoFisher Scientific Heraeus Pico 17, Osterode, Germany) for 15 min at 4 °C. Supernatants were collected and loading buffer was added to them. Protein samples were separated by sodium dodecyl sulfate-polyacrylamide gel electrophoresis (SDS-PAGE) and transferred to nitrocellulose, membranes. Then 5% nonfat milk in Tris-buffered saline-Tween (TBST) buffer was used to block them. The membranes were incubated with primary antibodies (Sangon Biotech, Shanghai, China) against Nrf2 and HO-1, later they were incubated with secondary antibody (anti-rabbit) (Promega, Beijing, China.) The membranes were visualized using enhanced chemiluminescence (ECL) (Kodak, Shanghai, China). Levels of protein were normalized to β-actin

### 4.6. Blood Lead Analysis

Acid digestion method was used for the preparation of blood sample, i.e., the blood sample was digested in nitric acid:hypochlorite (6:1) mixture and the lead level was estimated using a spectrophotometer (U-3900/3900H HITACHI, Eppendorf AG, Hamburg, Germany) at 620–650 nm wavelength. Statistical significance was evaluated by calculating standard deviation followed by Student’s *t*-test [39].

### 4.7. Reverse Transcription and Real-Time PCR

The expression of Nrf2, HO-1, GCL and GAPDH (Glyceraldehyde-3-phosphate dehydrogenase) were detected by reverse transcript and real-time PCR. Total RNA was extracted from the tissues using Trizol total RNA isolation reagent (Sangon Shanghai, China) as detailed by instructions from manufacturers. PCR (SYBR Premix Ex Taq, ROX Reference Dye, primers and cDNA template) (TaKaRa Biotechnology Dalian, China) were run in triplicates in 20 μL total reaction volume. The amplification conditions were as follows: 95 °C for 5 min, 39 cycles of 30 s at 95 °C, 60 °C for 30 s, 72 °C for 30 s and ended with extension temperature of 72 °C for 10 min. The fold changes were calculated on the basis of relative quantification method. The primers were designed as Nrf2 Fw 5′-CAGTGCTCCTATGCGTGAA-3′; Rv 5′-GCGGCTTGAATGTTTGTC-3′: HO-1 Fw 5′-ACAGATGGCGTCACTTCG-3′; Rv 5′-TGAGGACCCACTGGAGGA-3′: GCL Fw 5′-GGATGATGCCAACGAGTC-3′; Rv 5′-GTGAGCAGTACCACGAATA-3′: GAPDH Fw 5′-TCTGACGTGCCGCCTGGAGA-3′; Rv 5′-GGGGTGGGTGGTCCAGGGTT-3′.

### 4.8. Sperm Quality Evaluation

For normal testicular function, sperm count and morphology were considered as markers. Cauda epididymis was placed in Dulbecco’s modified Eagle’s medium, nutrient mixture F12 (DMEM/F12) with fetal bovine serum (FBS) 5% and cut into small pieces. The medium was balanced in incubator at temperature 37 °C and 5% CO_2_. Sperms from all four groups of mice were collected from their cauda epididymis and sperm number was determined by hemocytometer (Abcam Cambridge, MA, USA). Sperm morphology was also studied following a similar method with addition of eosin staining for a few minutes, then making a smear on a slide, air drying and mounting the slides for morphology study under microscope following the method of Majumder et al. [22] and WHO laboratory manual [40].

### 4.9. Fertility Performance

Adult ICR male mice were administered with LA and PU for four weeks, after which each of them was caged with untreated female ICR mice and provided with standard food. Males were separated from females after monitoring vaginal plugs within one week and if no plug was found still they were separated. Fertility performance of the four groups was analyzed on the basis of the time period when the females gave birth Pregnant female mice were inspected twice daily to appraise birth of offspring.

### 4.10. Statistical Analysis

Data represent mean ± standard error of the mean (SEM) of at least three independent experiments. One-way analysis of variance (ANOVA) was used for statistical comparison of groups and followed by Tukey-Kramer for a multiple comparisons test.

## Figures and Tables

**Table 1:** Effect of punicalagin (PU) on lipid peroxidation (LPO), glutathione GSH, 8-hydroxy-2′-deoxyguanosine (8-OHdG), superoxide dismutase (SOD) and catalase (CAT) in lead acetate (LA)- intoxicated mice testis (mean ± standard deviation (SD)).

	LPO (µmol/g Prot)	GSH (mg/g Prot)	8-OHdG (ng/mL Tissue Homogenate)	SOD (U/mg Prot)	CAT (nmol/mg Prot)
Control	1.93 ± 0.10	26.38 ± 0.90	1.16 ± 0.17	49.83 ± 0.98	72.91 ± 0.49
PU	1.95 ± 0.09	26.23 ± 0.96	1.18 ± 0.17	49.97 ± 1.17	72.97 ± 0.48
LA	10.45 ± 0.48	12.12 ± 1.38	6.70 ± 0.36	34.79 ± 0.52	52.83 ± 0.64
PU + LA	3.85 ± 0.35	22.14 ± 1.36	2.39 ± 0.32	47.92 ± 0.74	67.90 ± 0.49

**Table 2:** LA effect on testes weight grams (g), sperm abnormality (per 1000 sperm), blood lead level (µg/mL), sperm count (×10^6^). All results shown as mean ± standard error (SE).

	Group 1 (Control)	Group 2 (PU)	Group 3 (LA)	Group 4 (LA+ PU)
Blood lead level	0.17 ± 0.014	0.168 ± 0.01	0.426 ± 0.035 *	0.20 ± 0.022
Abnormal sperm	112.5 ± 1.85	112.3 ± 1.40	209 ± 4.4 *	125 ± 4.9
Testes weight	0.22 ± 0.02	0.216 ± 0.01	0.14 ± 0.01 *	0.198 ± 0.01
Sperm count	24 ± 1.03	23 ± 1.1	14.9 ± 0.9 *	20.6 ± 1.03

* *p* < 0.0001 as compared group 3 with group 1 and group 4.

**Table 3:** Mating test (mean ± SD).

	Control	PU	LA	PU + LA
No. of females pregnant	2 ± 0	2 ± 0	1.6 ± 1.03 *	2 ± 0
No. of pups	12 ± 0.92	12.2 ± 1.16	5.12 ± 4.45 *	10.25 ± 1.16

* *p* < 0.0001 as compared between control, LA, PU + LA groups.
